# Biological and antibacterial properties of the micro-nanostructured hydroxyapatite/chitosan coating on titanium

**DOI:** 10.1038/s41598-019-49941-0

**Published:** 2019-10-01

**Authors:** Baoe Li, Xiaomei Xia, Miaoqi Guo, Yu Jiang, Yu Li, Zhiyuan Zhang, Shimin Liu, Haipeng Li, Chunyong Liang, Hongshui Wang

**Affiliations:** 1 School of Materials Science and Engineering, Hebei University of Technology, Tianjin, 300130 China; 2 Department of Gem and Material Technology, Tianjin University of Commerce, Tianjin, 300134 China

**Keywords:** Atomic force microscopy, Atomic force microscopy

## Abstract

Titanium (Ti) is the widely used implant material in clinic, however, failures still frequently occur due to its bioinertness and poor antibacterial property. To improve the biological and antibacterial properties of Ti implants, micro-nanostructured hydroxyapatite (HA) coating was prepared on Ti surface by micro-arc oxidation (MAO), and then the antibacterial agent of chitosan (CS) was loaded on the HA surface through dip-coating method. The results showed that the obtained HA/CS composite coating accelerated the formation of apatite layer in SBF solution, enhanced cell adhesion, spreading and proliferation, and it also inhibited the bacterial growth, showing improved biological and antibacterial properties. Although, with the increased CS amount, the coverage of HA coating would be enlarged, resulting in depressed biological property, however, the antibacterial property of the composite coating was enhanced, and the cytotoxicity about CS was not detected in this work. In conclusion, the HA/CS coating has promising application in orthopedics, dentistry and other biomedical devices.

## Introduction

With the arrival of aging society, the number of people who suffered osteoarthritis, femoral head necrosis, bone defect and other bone diseases is dramatically increasing, resulting in a sharp rise in demand for bone repair and/or replacement by biomedical implants. Titanium (Ti) is the most widely used implant material, and it has been used successfully as hip joint, knee and tooth because of its small density, nontoxicity and good biocompatibility. However, the poor bioactivity and antibacterial property of Ti surface can cause loose fixation with bone tissues and postoperative infection, which limits its application^1,2^. Therefore, it is necessary to modify the Ti implant surface to improve its bioactivity and antibacterial property.

Currently, there are many methods to improve the bioactivity of Ti implant, such as preparing bioactive coatings (eg. hydroxyapatite (HA), titania and wallastone)^3^ and making surface patterns (microstructure, nanostructure and micro-nanostructure)^4^. Among the bioactive coatings, HA has the same inorganic composition as human bone tissue, so the HA coating is most frequently formed on Ti surface to improve the bioactivity. However, the methods to prepare HA coatings (such as sol-gel, plasma spray, and laser cladding, etc) usually have the shortcomings like long-period, high-cost, large complexity, and weak bonding strength between HA and Ti. As for the surface patterns, in our previous work, the micro-nanostructured surface has been proved to be a most suitable surface topography for implant material when compared with the other surface structures (eg. micro-structure, nanostructure), because it can mimic the structure of the natural bone tissues and change the mechanical force interaction at the cell-implant interface to stimulate bone formation^5,6^. Therefore, the ideal bioactivity can be designed for Ti surface by forming a micro-nanostructred HA coating which has strong bonding strength with Ti substrate through a simple and economical method.

Micro-arc oxidation (MAO) is a simple and efficient electrochemical method for metal surface treatment with low cost. During the MAO process, spark and discharge spots are generated on the metal surface in electrolyte solution, and consequently form the ceramic coatings with different structures under the synthetical effect of thermochemistry, plasma chemistry and electrochemistry^7^. Because the MAO induced ceramic coatings are partial conversion of the body material, there is no sharp interface between the substrate and the oxide layer, their bonding strength and stability have been proved very high and can be used safely and stably in the service environment^8,9^. Till now, some researchers have prepared HA coatings on Ti surface by MAO to improve bioactivity, however, the coating structure, especially the HA coatings with micro-nanostructure, has been seldom explored.

To improve the antibacterial property, antibiotics could be introduced to the implant surface to decrease the risk of postoperative infection by preventing the bacterial adhesion and proliferation. In recent years, the antibacterial agent of chitosan (CS) has been widely studied and applied^10^. Compared to the inorganic bacterial agent (eg. Ag), CS shows more advantages due to its nontoxicity, favorable biocompatibility and biodegradation^11^. Some researchers have explored the combination of HA and CS to achieve good bioactivity and proper antibacterial property^12–16^. For example, Pang prepared electrolyte by mixing HA powders with positive charged CS solution, and then prepared HA/CS composite coating by electrophoretic deposition method^15^. Redepenning used a two-step method to prepare HA/CS composite coating^16^. Firstly, the composite coating of brushite/CS was prepared by electrochemical deposition, and then the coating was soaked in sodium hydroxide solution and converted into HA/CS. Although they can achieve good bioactivity and antibacterial property, the former method may lead to uneven dispersion of HA particles in the coating, and the latter may lead to incomplete conversion of brushite into HA. In addition the bonding strength between composite coatings and substrate is always unsatisfied. Up to now, there has been no reports of loading CS on the HA coatings which was prepared beforehand on Ti surface.

Therefore, in this work, micro-nanostructred HA coating was first prepared on Ti surface by MAO, and then the antibacterial agent of CS was loaded on the surface of HA. It was expected that the HA/CS coating would endow the Ti implant with good bioactivity and antibacterial property. The biological property was investigated by simulated body fluid (SBF) soaking test and MC3T3-E1 cell culture experiment. The antibacterial effect against Escherichia coli (*E. coli*) was evaluated by the bacterial counting method, zone of inhibition (ZOI) test and optical technique.

## Materials and Methods

### Sample preparation

Pure Ti sheets (TA1, Xi’an, China) in dimensions of 10 mm × 10 mm × 1 mm were polished with 800#, 1000#, 1500#, and 2000# sandpapers until no obvious scratch can be observed on the surface, and then ultrasonically cleaned respectively in acetone, ethanol and deionized water for 3 min, and dried in air.

The MAO process was carried out in an electrolyte containing 0.2 mol/L calcium acetate ((CH_3_COO)_2_Ca·H_2_O) and 0.1 mol/L monosodiumorthophosphate (NaH_2_PO_4_·2H_2_O) at 390 V for 3 min using a direct current (DC) power supply. The applied pulse frequency and duty cycle were separately 100 Hz and 50%. The Ti sheet was used as anode, platinum plate as cathode, and the distance between the two electrodes was 5 cm. After the MAO treatment, the samples were cleaned with deionized water and referred as HA coating. For CS loading, the HA coating was soaked into the 2% acetic acid solutions with different CS concentrations (0.2, 0.5, 1, 2, 4, 6 mg/ml) for 3 min, and then rinsed with deionized water for 5 seconds, and dried in an oven at 55 °C for 24 hours. The obtained samples were respectively named HA/0.2CS, HA/0.5CS, HA/1CS, HA/2CS, HA/4CS, HA/6CS.

### Surface characterization

The surface morphologies and elemental composition of the obtained samples were separately examined by field emission electron microscope (SEM, American FEI, Nova Nano SEM450) and the energy dispersive X-ray spectrometer (EDS). The phase composition of the surface coatings was analyzed by X-Ray Diffraction (XRD, Rigaku D/max2500) and fourier transferinfrared spectroscopy (FT-IR, V80, German). Thermogravimetric analysis (TGA) was used to measure the amount of the loaded HA and CS on Ti surface, atomic force microscope (AFM, Agilent 5500) was used to detect the surface roughness, and contact angle meter (OCA30) was used to evaluate the hydrophilicity from the measurements of the contact angle between deionized water and the sample surface at room temperature.

### Evaluation of biological property

The bioactivity of the obtained samples was evaluated by investigating its capability to induce bone-like apatite formation in SBF at 37 °C. The experimental process was described in detail elsewhere^17^. The growth of apatite on sample surfaces was investigated by SEM and XRD.

The biocompatibility was evaluated by *in vitro* cell culture experiment. The MC3T3-E1 cells (the Cell Bank of Type Culture Collection of Chinese Academy of Sciences, China) were seeded on the sterilized sample surfaces at a density of 7 × 10^4^ cells/cm^2^, and cultured in α-modified eagle medium (Gibco) containing 10% fetal bovine serum and 3% penicillin streptomycin at 37 °C in a saturated humid atmosphere containing 5% CO_2_. After culturing for 1, 4 and 7 days, the cell proliferation was analyzed by Cell Counting Kit-8 (CCK-8, Dojindo, Kumamoto, Japan). And then the attached cells which were cultured for 4 days were washed with phosphate-buffered saline (PBS, pH = 7.4) and soaked overnight in 2.5% glutaraldehyde. After dehydrated in a grade ethanol series and hexamethyldisilazane (HMDS) according to the literature^18^, the cell morphologies were observed by SEM.

### Evaluation of antibacterial property

The antibacterial property of the HA/CS coatings against *E. coli* was examined by bacterial counting method, ZOI test and optical technique.

For bacterial counting method, 20 μl solution containing 1 × 10^5^ cfu/ml *E. coli* was introduced onto the sample surface and incubated at 37 °C for 24 h. After incubation, the bacteria on the sample surface was detached by ultrasonic cleaning with normal saline. The bacteria in the saline solution was re-cultivated on agar plates to count the colonies according to the ref.^19^. For ZOI test, 10 μl *E. coli* with the concentration of 1 × 10^7^ cfu/ml was spread evenly over Mueller-Hinton plates, and then samples were placed on it and incubated for 24 h at 37 °C, finally photographed to record the result. While for the optical technique, the samples were incubated in 5 ml bacteria suspension with a concentration of 1 × 10^4^ cfu/ml for 24 h, and then the viable bacteria in the suspension was evaluated by ultraviolet spectrophotometry. To investigate the long-lasting antibacterial effect, the samples were also incubated in 20 μl bacteria suspension (containing 1 × 10^5^ cfu/ml *E. coli*) for 48 h, 96 h and 144 h, and then they were cleaned and sterilized by ultraviolet irradiation for 24 h, and their antibacterial effect was examined by bacterial counting method again. The antibacterial rate was determined by the following equation:

$$\begin{array}{ccc}{\rm{A}}{\rm{n}}{\rm{t}}{\rm{i}}{\rm{b}}{\rm{a}}{\rm{c}}{\rm{t}}{\rm{e}}{\rm{r}}{\rm{i}}{\rm{a}}{\rm{l}}\,{\rm{r}}{\rm{a}}{\rm{t}}{\rm{e}}({\rm{ \% }}) & = & ({\rm{C}}{\rm{F}}{\rm{U}}\,{\rm{o}}{\rm{f}}\,{\rm{H}}{\rm{A}}\,{\rm{c}}{\rm{o}}{\rm{n}}{\rm{t}}{\rm{r}}{\rm{o}}{\rm{l}}-{\rm{C}}{\rm{F}}{\rm{U}}\,{\rm{o}}{\rm{f}}\,{\rm{H}}{\rm{A}}/{\rm{C}}{\rm{S}})/\\  &  & \,{\rm{C}}{\rm{F}}{\rm{U}}\,{\rm{o}}{\rm{f}}\,{\rm{H}}{\rm{A}}\,{\rm{c}}{\rm{o}}{\rm{n}}{\rm{t}}{\rm{r}}{\rm{o}}{\rm{l}}\times 100{\rm{ \% }}.\end{array}$$

### Statistical analysis

Experiments were run in triplicate per sample. Standard deviations were plotted as error bars for the data points on all figures. Statistically significant differences were assessed by SAS. Difference with p values < 0.05 was considered to be significant.

## Results and Discussion

**Figure 1:**
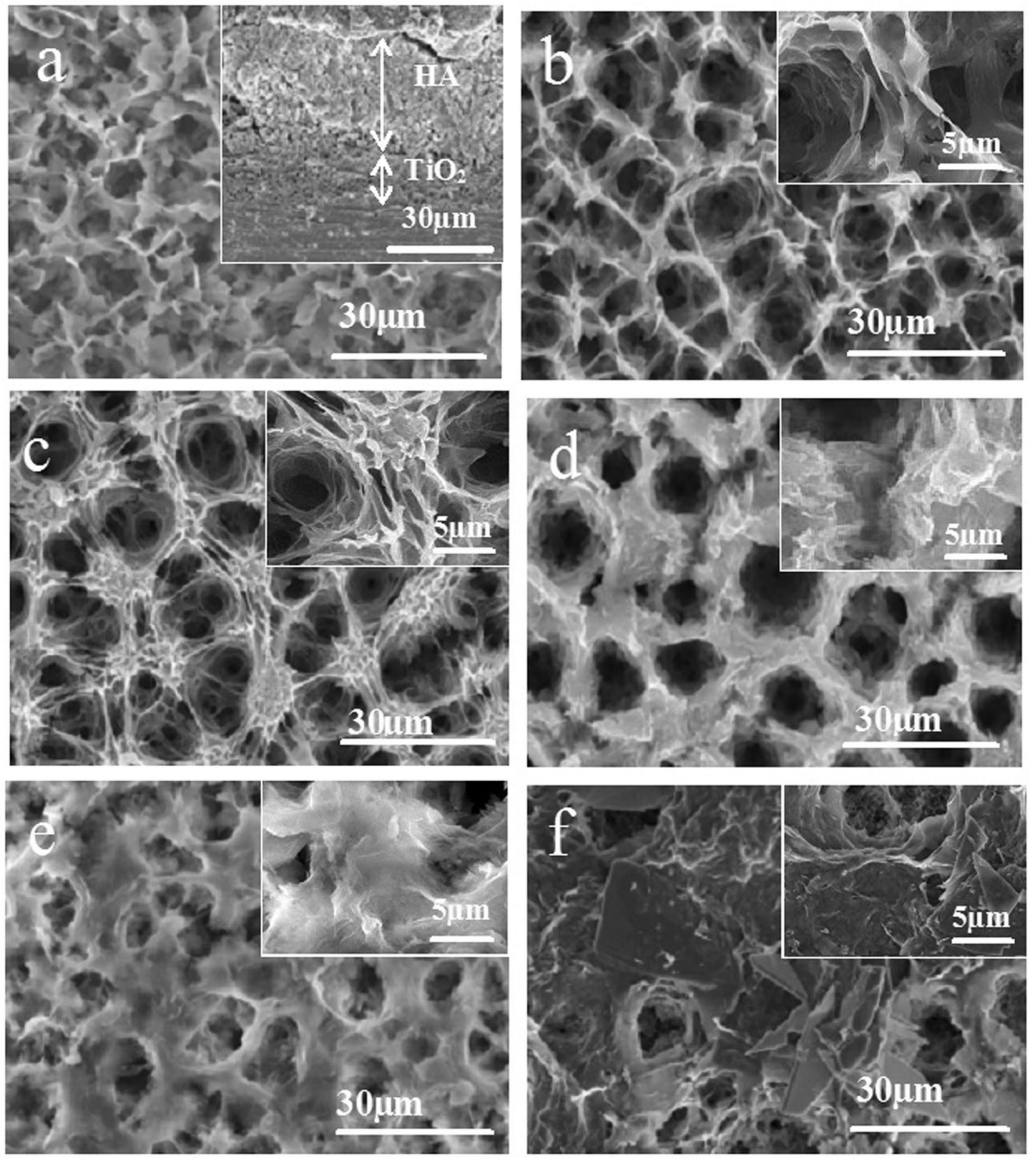
Surface morphologies of the HA and HA/CS composite coating: HA (**a**), HA/0.2CS (**b**), HA/0.5CS (**c**), HA/1CS (**d**), HA/2CS (**e**), HA/6CS (**f**).

Figure 1 showed the surface morphologies of the HA and HA/CS coatings. For HA coating (Fig. 1a), it can be seen that a porous micro-nanostructure was formed on Ti surface by MAO. The diameter of the micro-pores was 3 ± 1 μm, and the porosity was about 1 ± 0.3%. The micro-pores were a consequence of electric breakdown and surface reconstruction on the weak parts of Ti surface during MAO. Around the micro-pores, a petaling substance was observed. Analyzing the structure by the image processing software (Nano Measurer 1.2), the petals were about 1 ± 0.1 μm in width and 2 ± 0.4 μm in length, vertically oriented and randomly distributed on Ti surface and clustered to form some nanostructures. The petal density was 8 ± 2 petals/100 μm^2^ and the average space between petal sheets was 2 ± 1 μm. From the cross-section (top right corner of Fig. 1a), it can be seen that the thickness of the HA coating was 29 ± 2 μm, and there is no delamination between coatings. Measured by scratch method, the bonding strength between HA and TiO_2_ was 16 ± 2 N, and 28 ± 1 N between TiO_2_ and Ti substrate, confirming the strong connection between coatings. After CS loading on the HA coating, it can be seen that, the original HA coating was covered to a certain degree. When the concentration of CS solution was 0.2 mg/ml, the skeleton of the original micro-nanostructure of HA coating still can be observed (Fig. 1b). As the CS concentration increased to 0.5 mg/ml, the petaling structure cannot be seen clearly (Fig. 1c). When the concentration increased to 1 mg/ml, only micro-pores were visible (Fig. 1d). The CS concentration of 2 mg/ml can even cover the micro-pores of HA coating (Fig. 1e). As the CS concentration was further increased to 4 mg/ml and 6 mg/ml, only the smooth CS film can be observed on Ti surface (Fig. 1f). However, the influence of CS on coating structures was not observed in the cross-section images (figures omitted).

The elemental composition of the corresponding coating surfaces was listed in Table 1. All the coatings contained elements like calcium (Ca), phosphorus (P), oxygen (O) and titanium (Ti). The content of Ti was relatively low, and the content of Ca, P and O was much higher, which demonstrated that the Ti substrate was covered by a Ca-P coating after MAO treatment. The Ca/P value (atomic ratio) was about 1.6, similar to the Ca/P value in HA, indicating the formation of HA on Ti surface. After CS loading, the elements of carbon (C) and nitrogen (N) were also found in the HA/CS composite coatings. Because C and N are the main elements of CS molecules, the EDS results indicated that a CS film was successfully loaded on the surface of HA coating. In addition, as the concentration of CS solution increased, the detected amount of C and N gradually increased, meaning that the amount of CS loaded on the HA coating increased with the concentration of CS solution.

**Table 1:** EDS results of the elements in HA and HA/CS coatings (At.%).

Sample	Ca	P	Ti	O	C	N
HA	22.28	13.73	0.43	62.92	0.64	0
HA/0.2CS	19.70	12.94	4.02	56.44	6.47	0.41
HA/0.5CS	19.03	11.58	2.87	57.76	9.01	0.74
HA/1CS	18.32	10.91	1.12	55.96	11.08	1.78
HA/2CS	17.45	9.98	0.30	56.59	13.34	2.34
HA/4CS	15.61	9.04	0.14	55.63	17.43	3.14
HA/6CS	13.83	7.48	0.19	54.09	18.90	5.51

**Figure 2:**
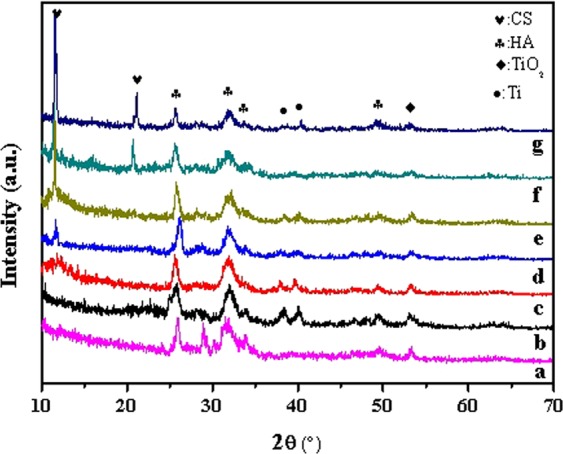
XRD patterns of the HA and HA/CS coatings: HA (**a**), HA/0.2CS (**b**), HA/0.5CS (**c**), HA/1CS (**d**), HA/2CS (**e**), HA/4CS (**f**), HA/6CS (**g**).

**Figure 3:**
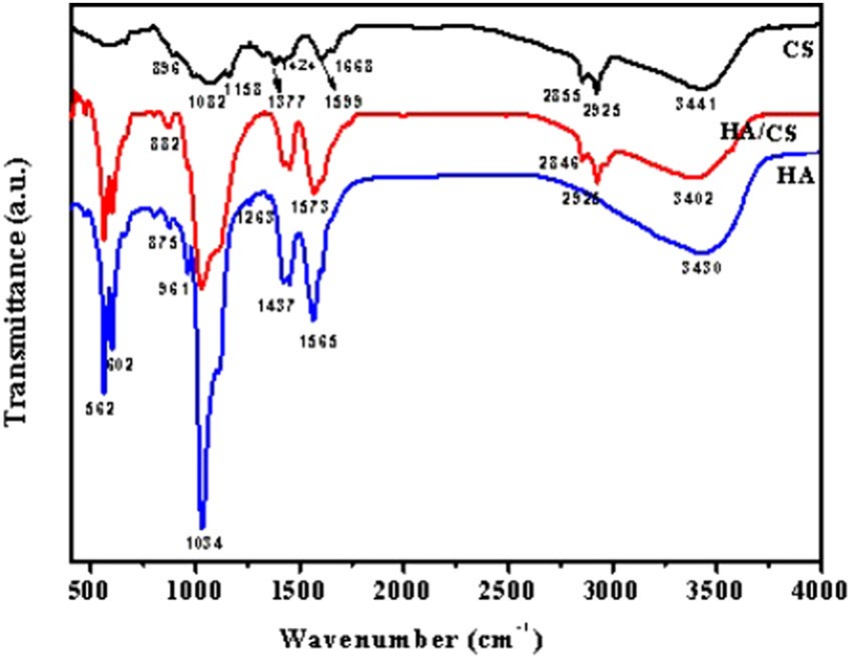
FT-IR spectra of the HA, CS and HA/0.2CS composite coating.

Figure 2 exhibited the XRD spectra of the HA and different HA/CS coatings. For HA coating, the diffraction peaks representing HA were detected at 2theta 26°, 32°, 34° and 50°^9^, and confirmed the successful preparation of HA on Ti surface by MAO. Although the diffraction peaks representing titania at 53° and 64° were also observed^8^, the low peak intensity indicated that the HA coating has a large thickness and almost covered the titania which was definitely formed during the MAO process. The XRD pattern of HA coating showed good agreement with the literature^20^, and in the literature, the formation process of HA and titania had been described in detail. After loading of CS, the typical peaks for CS can be detected at 2theta of 12° and 21° ^21^. However, when the CS concentration was low (0.2 mg/ml, 0.5 mg/ml), the peak strength of CS in composite coatings was weak, which may result from the low amount of CS on HA coatings (however, the existence of CS was proved by FTIR and shown in Fig. 3). From the XRD spectra, it can also be found that after CS loading on HA surface, the intensity of typical HA peaks all decreased slightly, which proved again that the CS layer was formed on the surface of HA coating and covered the original HA and titania to an extent. The existence of Ti was also detected in some coatings, which may be due to that the composite coating was partially peeled and the substrate was exposed outside. The XRD spectra were in good agreement with the surface morphologies shown in Fig. 1 and the EDS results of Table 1.

Figure 3 showed the FT-IR spectra of HA, CS and HA/0.2CS coatings. For HA coatings, the peaks standing for PO_4_^3−^ (562 cm^−1^, 602 cm^−1^, 961 cm^−1^) and -OH (1565 cm^−1^, 3430 cm^−1^) were observed, confirming the existence of HA on surfaces. For CS, the main functional groups of CS were also detected, the peaks at 1377 cm^−1^ and 1424 cm^−1^ represent -CH_3_ group, the peaks at 2855 cm^−1^ and 2925 cm^−1^ represent C-H group. Besides, -CHOH- (1082 cm^−1^), C-O-C (1158 cm^−1^), -NH_2_ (1599 cm^−1^) and -OH (3441 cm^−1^) groups representing CS were also detected. After loading CS on HA coating (HA/0.2CS composite coating), the peaks standing for HA and CS were both observed, confirming the existence of CS on the surface of HA coating. However, the peaks of -NH_2_ and -OH both shifted to the direction of low wave number, which might be caused by hydrogen bonds between CS and HA.

**Figure 4:**
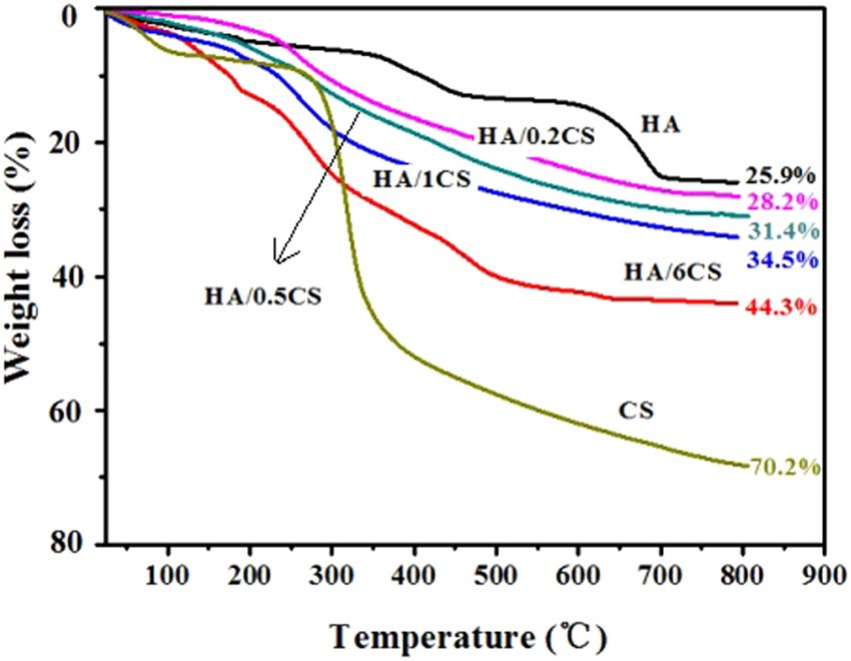
TGA curves of the different samples.

To measure the effective amount of HA and CS on Ti surface, the samples were examined by TGA, and the results were shown in Fig. 4. It can be seen, CS showed the largest weight loss, HA/CS coatings showed less, and HA showed the least weight loss. With the increase of CS amount on HA surface, the weight loss was enhanced. If X and Yn (n is 0.2, 0.5, 1, 6) respectively represent the weight (mg) of HA and CS in the different HA/CS composite coatings (per unit area), based on the TGA curves, the following equations can be obtained,

$$\begin{array}{ll}{\rm{For}}\,{\rm{HA}}/0.2{\rm{CS}}: & (25.9 \% \,\ast \,{\rm{X}}+70.2 \% \,\ast \,{\rm{Y}}0.2)/({\rm{X}}+{\rm{Y}}0.2)=28.2 \% \\ {\rm{For}}\,{\rm{HA}}/0.5{\rm{CS}}: & (25.9 \% \,\ast \,{\rm{X}}+70.2 \% \,\ast \,{\rm{Y}}0.5)/({\rm{X}}+{\rm{Y}}0.5)=31.4 \% \\ {\rm{For}}\,{\rm{HA}}/1{\rm{CS}}: & (25.9 \% \,\ast \,{\rm{X}}+70.2 \% \,\ast \,{\rm{Y}}1)/({\rm{X}}+{\rm{Y}}1)=34.5 \% \\ {\rm{For}}\,{\rm{HA}}/6{\rm{CS}}: & (25.9 \% \,\ast \,{\rm{X}}+70.2 \% \,\ast \,{\rm{Y}}6)/({\rm{X}}+{\rm{Y}}6)=44.3 \% \end{array}$$

Consequently, the amount of HA and CS on Ti surface can be expressed as:

$${\rm{Y}}0.2=2.3{\rm{X}}/42,\,{\rm{Y}}0.5=5.5{\rm{X}}/38.8,\,{\rm{Y}}1=8.6{\rm{X}}/35.7,\,{\rm{Y}}6=18.4{\rm{X}}/25.9$$

Considering that the effective mass of HA on Ti surface was 6.802 mg/cm^2^ (namely, X = 6.802, based on the TGA curve), the CS amount on different HA/CS coatings was obtained.

$${\rm{Y}}0.2=0.372\,{\rm{mg}}/{{\rm{cm}}}^{2},{\rm{Y}}0.5=0.964\,{\rm{mg}}/{{\rm{cm}}}^{2},{\rm{Y}}1=1.639\,{\rm{mg}}/{{\rm{cm}}}^{2},{\rm{Y}}6=4.834\,{\rm{mg}}/{{\rm{cm}}}^{2}$$

It was clear that, on HA surface, the loaded CS amount increased with the CS concentration, in good agreement with the morphologies shown in Fig. 1.

**Figure 5:**
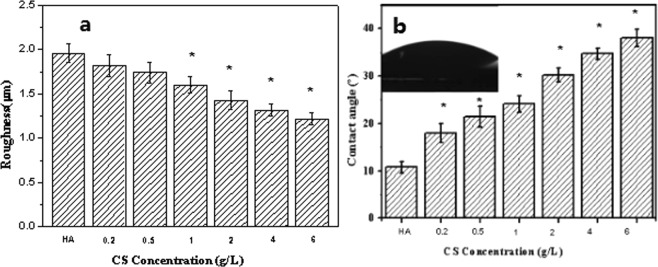
Surface roughness (**a**) and water contact angles (**b**) of the corresponding composite coatings, data expressed as mean ± SD (n = 3), *a statistical significance compared to the HA group (P < 0.05).

Figure 5 showed the surface roughness and hydrophilicity of the HA and HA/CS composite coatings. It can be seen that, after MAO treatment, the HA coating showed a surface roughness in micro scale. However, due to the existence of nanostructured HA patals, the coating showed excellent hydrophilicity. When CS was introduced to the surface of HA coating, the roughness and hydrophilicity were both decreased. And with the increase of the loaded CS amount, the roughness and hydrophilicity decreased rapidly, which may be due to that the coverage of the original HA coating was gradually enhanced as the loaded CS amount was increased. However, the HA/CS coating remained hydrophilic and the surface roughness was still in micro scale even when the CS concentration was increased to 6 mg/ml, indicating the good hydrophilicity and homogeneity of the CS membrane. Based on the literature^22^, the good hydrophilicity of CS was attributed to the synergistic effect of the following cases: the protonation induced -NH_3_^+^ in CS molecules can attract OH^−^ in H_2_O molecules via hydrogen bond, the functional groups of -CH_2_ on the side chain of CS can also form hydrogen bond with H_2_O molecules, and the CS molecule also contains a large number of hydroxyl groups. As the CS amount was increased, the hydrophilicity of the coating surface decreased gradually, because the hydrophilicity was not only determined by the composition but also the nanostructure and surface area. As the CS amount was increased, the nanostructures of HA petals was covered, and the surface area was decreased, which can be observed in Fig. 1.

**Figure 6:**
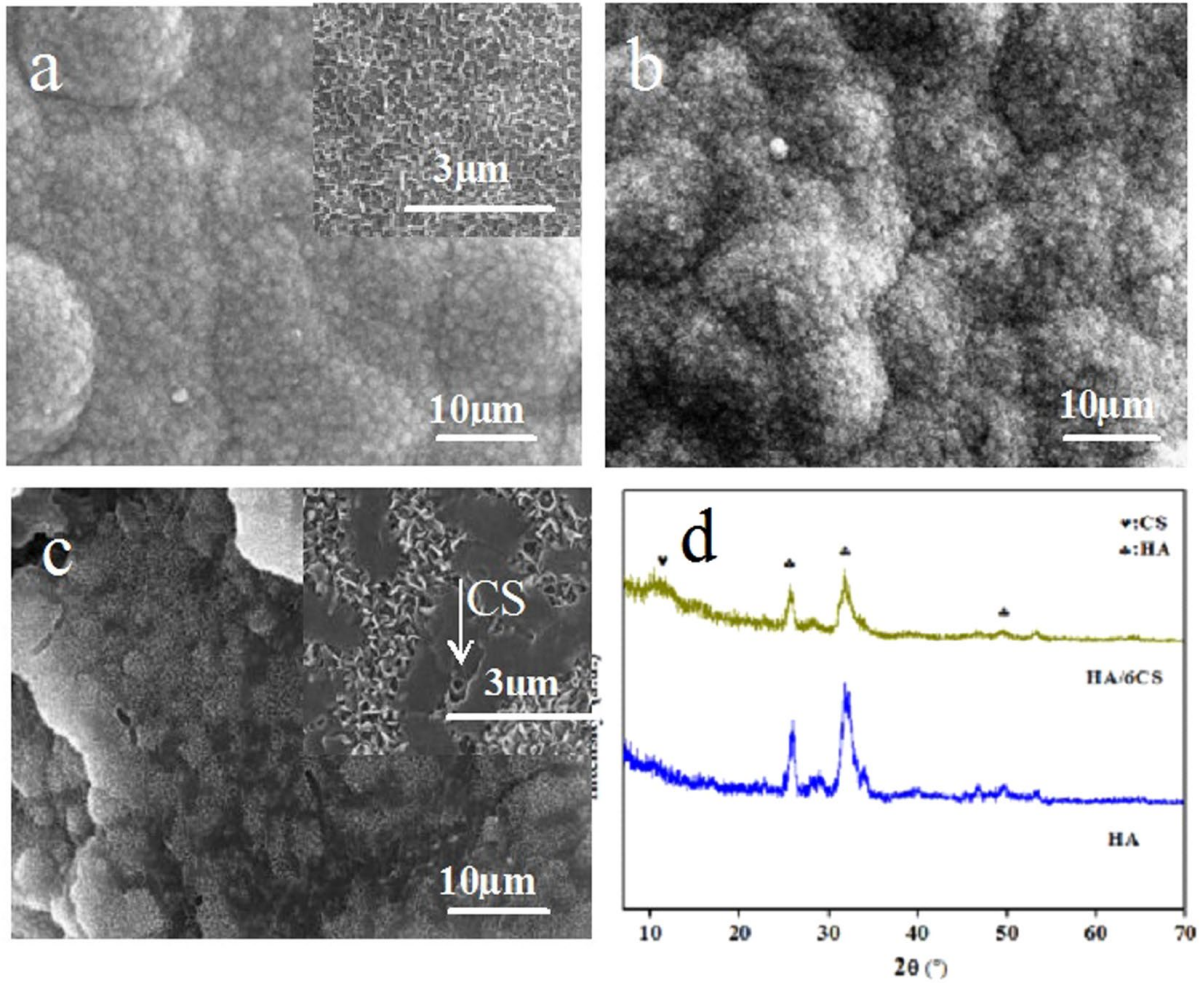
Surface morphologies of HA (**a**), HA/0.2CS (**b**), HA/6CS (**c**) coatings after immersion in SBF for 2 weeks, and corresponding XRD patterns of the HA and HA/6CS coatings (**d**).

Surface roughness and hydrophilicity are well known key factors that determine the biological properties such as apatite formation in SBF, cell adhesion and proliferation. In this work, the obtained composite coatings all showed large surface roughness and hydrophilicity, and they were expected to have good biological properties. Figure 6 showed the surface morphology of the coatings after soaking in SBF for 2 weeks. It can be seen that, large amount of apatite sheets were formed on the surfaces of HA and HA/0.2CS coatings, and the new apatite sheets grew into a compact layer. The original micro-nanostructure of HA petals was totally covered by the new grown apatite, indicating the good bioactivity. With the increase of loaded CS amount, the new formed apatite sheets decreased slightly. On the surface of the HA/6CS coating, the least apatite sheets were formed, and the loaded CS can still be observed on the surface.

Table 2 listed the element composition of the different coatings. It can be seen that after soaking in SBF for 2 weeks, the Ca and P content increased significantly when compared with those before soaking, confirming that the coatings were all conducive to the deposition of Ca and P. Because the Ca/P ratio was close to the 1.67, the formation of apatite was proved again (in good agreement with the XRD pattern of Fig. 6d). With the increase of CS amount, the content of Ca and P gradually decreased, which was also consistent with the surface morphology of Fig. 6. The SBF soaking test revealed that the CS film was also conducive to apatite formation, however, its bioactivity was inferior to HA. The bioactivity of CS was displayed because a large number of protonated amino groups on CS surface can absorb OH- in SBF via hydrogen bond and electrostatic attraction, which consequently would adsorb the Ca^2+^ and PO_4_^3−^ in solution by electrostatic attraction, and thus finally form the bone-like apatite. In addition, the CS molecule also contains a large amount of -OH, which itself is the nucleate site of HA^23^. While, the HA coating is constituted of Ca, P and -OH, which can directly react with the ions in SBF to form apatite rapidly. When compared with the CS film, the HA coating showed more nucleation sites of apatite within the same surface area, so it can accelerate the formation of apatite faster in SBF.

**Table 2:** EDS results of the elements in HA and HA/CS coatings after immersion in SBF for 2 weeks (At.%).

Sample	C	N	O	Ca	P	Ti
Ti	3.32	0	17.39	0.37	0.24	78.68
TiO_2_	3.14	0	62.32	10.09	5.98	18.44
HA	8.13	0	47.58	27.35	16.94	0
HA/0.2CS	11.41	2.74	44.16	25.87	15.82	0
HA/6CS	16.67	5.72	51.43	16.6	9.58	0

**Figure 7:**
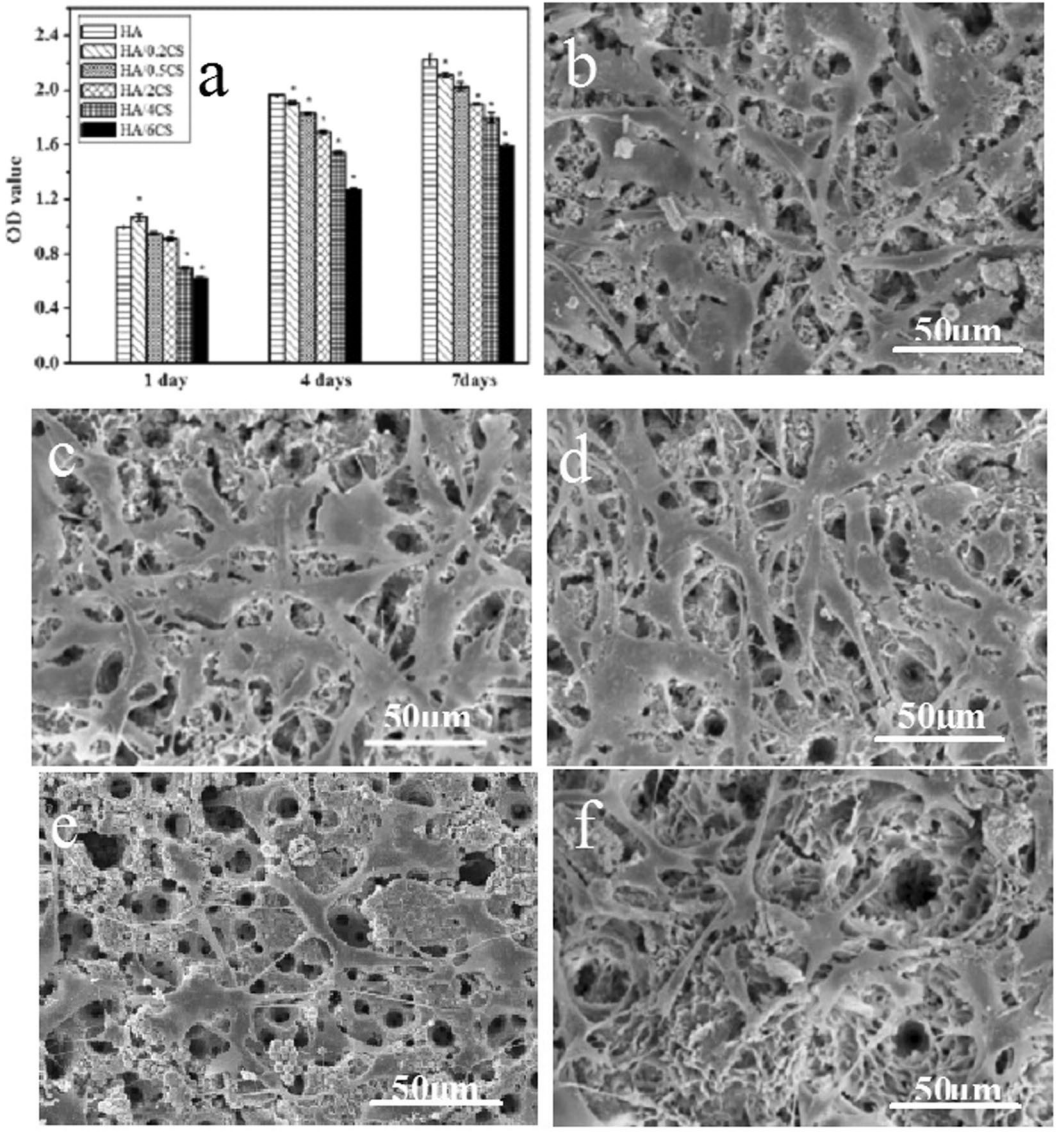
CCK-8 results representing the MC3T3-E1 cell proliferation on the different coatings, data expressed as mean ± SD (n = 5), *a statistical significance compared to the HA group (P < 0.05) (**a**), and cell morphologies on HA (**b**), HA/0.2CS (**c**), HA/0.5CS (**d**), HA/2CS (**e**), HA/6CS (**f**) after culturing for a day.

Figure 7 showed the cell morphologies and proliferation rate on the different coating surfaces. During the cell culture period, a progressive and significant increase in cell numbers can be detected on all coatings. However, the cells proliferated much faster on the HA coating than those on the HA/CS coatings (p < 0.05), and the cell number on HA/CS coatings decreased with the increase of CS concentration, which may be attributed to the coverage of micro-nanostructured HA coating by CS. However, the cell morphologies turned out that all the coating surfaces benefited the cell adhesion and spreading. Cells showed a typical polygonal osteoblastic shape. The abundant finger-like protrusions and filopodia stretched out from the cell body, and no significant difference in cell morphology can be observed during this experimental period. The results confirmed that all the coatings showed excellent biocompatibility, and no cytotoxicity of CS can be observed.

**Figure 8:**
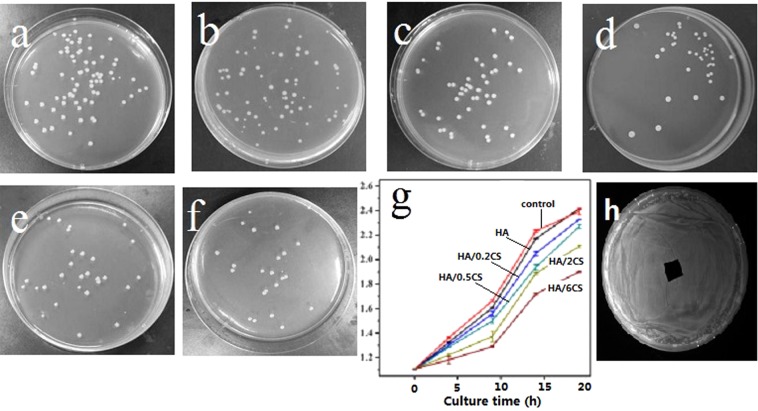
Bacterial colony of different samples for cultivated in bacteria solution for 24 hours: HA (**a**), blank control **(b**), HA/0.2CS (**c**), HA/0.5CS (**d**), HA/2CS (**e**), HA/6CS (**f**). The absorbance value representing the antibacterial effect (data was expressed as mean ± SD (n = 3), a statistical significance can be seen when compared to the control and HA groups) (**g**), and the ZOI test result of HA/6CS (**h**).

**Figure 9:**
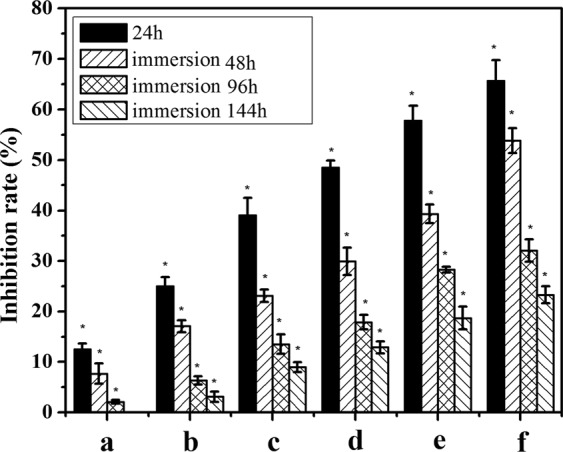
The antibacterial rate of different surface coatings after cultivated in bacteria solution for 24, 48, 96 and 144 hours (HA coating as the control sample): HA/0.2CS (**a**), HA/0.5CS (**b**), HA/1CS (**c**), HA/2CS (**d**), HA/4CS (**e**) and HA/6CS (**f**). Data expressed as mean ± SD (n = 3), *a statistical significance compared to the HA group (P < 0.05).

The antibacterial effect of the HA and HA/CS coatings was assessed by bacterial counting method, ZOI test and optical technique, as shown in Fig. 8. It can be seen that the growth of *E. coli* was scarcely inhibited on the HA coating (Fig. 8a), while the number of bacterial colonies grown on HA/CS coatings was much less, and it decreased with the increase of loaded CS amount (Fig. 8c–f). The antibacterial effect of HA/CS was also confirmed by the optical technique (Fig. 8g). The absorbance value of the vertical axis represented the number of bacterial colonies. The absorbance value of HA coating was the highest. With the increase of CS amount, the absorbance value decreased, which is due to that the increased CS amount enhanced the antibacterial performance. However, the bacteriostatic rings were not observed in this work, even though the maximum amount of chitosan was used (Fig. 8h), which indicated that, to maintain the bactericidal effect, CS need to destroy the membrane of bacteria by direct contact with them. The disappearance of bacteriostatic ring also proved that CS was bonded firmly on HA surface, which benefit achieving the long-term antibacterial effect. The long-term antibacterial effect of different surface coatings was also investigated by bacterial counting method, and the antibacterial rates were summarized and shown in Fig. 9. It can be seen that, for different surface coatings, the antibacterial rate decreased with the increase of culture time. After cultured for 144 h, HA/0.2CS even lost its antibacterial property completely. The antibacterial effect was prolonged as the loaded CS amount was increased. When the degradation rate is constant, more CS amount would result in longer degradation time, thus the longer antibacterial effect was maintained.

The antibacterial mechanism of CS has been reported in the literature. The protonated CS can induce electrostatic attraction to the negatively charged bacteria, and form a dense polymer membrane on the surface of the cell, preventing the transport of nutrients to the cell and the discharge of physiological metabolic waste, resulting in the metabolic disorder of bacteria, and inhibiting the growth and reproduction of bacteria. In addition, the protonated amino group can also cause the negative charge to be disordered on the cell wall and the cell membrane, making it difficult to form the cell wall when growing. As a result, the bacterial lysis and the rupture of the cell membrane would occur due to the great osmotic pressure of the outside environment, which also made the inhibition of CS to *E. coli* more remarkable^24^.

In this work, the HA/CS composite coatings with different CS concentrations were prepared on Ti. However, it was found that the increase of CS amount would depress the bioactivity of HA to some extent, although it could increase the antibacterial property. The depressed bioactivity was due to the coverage of HA coating and its micro-nanostructure by CS membrane. Therefore, to achieve ideal bioactivity and antibacterial property, the loaded CS amount should be properly controlled. In this research work, CS solutions of 0.2 mg/ml and 0.5 mg/ml were considered to be appropriate. However, the optimization of the loaded CS amount, the degradation rate and long-term antibacterial effect of the CS membrane still need to be investigated in detail in the future work.

## Conclusions

In this work, micro-nanostructured HA coating was prepared on Ti surface by MAO, and then the antibacterial agent of CS was introduced to the HA surface by dip-coating method to achieve good biological and antibacterial property. The loaded CS amount can be adjusted by controlling the concentration of CS solution. As the loaded CS amount was increased, the biological property would be depressed to a certain degree, however, the antibacterial property would be enhanced. The depressed biological property was attributed to the coverage of HA and micro-nanostructure. By controlling the loaded CS amount, the HA/CS composite coating would endow Ti implant with improved biological and antibacterial properties.
